# Phenotypic and genetic characterization of resistance in *Arabidopsis thaliana* to the oomycete pathogen *Phytophthora parasitica*

**DOI:** 10.3389/fpls.2015.00378

**Published:** 2015-05-27

**Authors:** Yuling Meng, Yihua Huang, Qinhu Wang, Qujiang Wen, Jinbu Jia, Qiang Zhang, Guiyan Huang, Junli Quan, Weixing Shan

**Affiliations:** ^1^College of Plant Protection, Northwest A&F UniversityYangling, China; ^2^State Key Laboratory of Crop Stress Biology for Arid Areas, College of Plant Protection, Northwest A&F UniversityYangling, China; ^3^College of Life Sciences, Northwest A&F UniversityYangling, China

**Keywords:** *Arabidopsis thaliana*, *Phytophthora parasitica*, resistance, Zu-1, genetic analysis

## Abstract

The interaction between *Arabidopsis thaliana* and the oomycete pathogen *Phytophthora parasitica* emerges as a model for exploring the molecular basis and evolution of recognition and host defense. Phenotypic variation and genetic analysis is essential to dissect the underlying mechanisms in plant–oomycete interaction. In this study, the reaction phenotypes of 28 *A. thaliana* accessions to *P. parasitica* strain Pp016 were examined using detached leaf infection assay. The results showed the presence of four distinct groups based on host response and disease development. Of all the accessions examined, Zurich (Zu-1) is highly resistant to *P. parasitica*. Microscopic characterization showed that rapid and severe hypersensitive response at the primary infection epidermal cells is associated with disease resistance. Furthermore, Zu-1 is resistant to a set of 20 diverse *P. parasitica* strains, which were collected from different host plants and exhibited differential specificities on a set of tobacco cultivars. However, Zu-1 is susceptible to *P. parasitica* when the root is inoculated, suggesting differential expression of associated resistance genes in the root and foliar tissues. Genetic analysis by crossing Zu-1 and the susceptible accession Landsberg (Ler) showed that the resistance in Zu-1 to *P. parasitica* is semi-dominant, as shown by infection assays of F_1_ progenies, and is likely conferred by a single locus, defined as *RPPA1^*Zu-1*^* (for Resistance to *P. parasitica* 1), as shown by analysis of F_2_ segregating populations. By employing specific-locus amplified fragment sequencing (SLAF-seq) strategy to identify molecular markers potentially linked to the locus, the strongest associated region was determined to be located between 7.1 and 11.2 Mb in chromosome IV. The future cloning of *RPPA1^*Zu-1*^* locus will facilitate improved understanding of plant broad-spectrum disease resistance to oomycete pathogens.

## Introduction

Oomycetes represent a group of eukaryotic microorganisms related to diatoms and brown algae, causing many destructive diseases to plants and animals (Beakes et al., 2012). Among the group, *Phytophthora* is the best-studied genus that includes over 100 species, which are divided into 10 clades (Kroon et al., 2012). *Phytophthora parasitica* Dastur (syn. *P. nicotianae* Breda de Haan) is classified in the *Phytophthora* clade 1 and its closest relatives include *P. infestans* (Blair et al., 2008; Kroon et al., 2012). Unlike the well-studied species *P. infestans*, which is a foliage pathogen and only infected few plants, *P. parasitica* is a typical root pathogen with a broad-range of host plants, being capable of infecting over 72 plant genera (Meng et al., 2014). The two species vary in genome sizes, being 82 and 240 Mb for *P. parasitica* and *P. infestans*, respectively (Judelson, 2012). However, nearly half of *Phytophthora* species are mostly pathogenic on roots, and about 30% species being pathogens of multiple host plants (Kroon et al., 2012). Therefore, *P. parasitica* provides an opportunity for their role in understanding plant recognition and infection, and their broad host ranges. Moreover, while being a natural pathogen of tobacco species, *P. parasitica* is capable of infecting the model plant species *Arabidopsis thaliana*, which allows accelerated understanding of *Phytophthora* pathogenesis and plant susceptibility (Attard et al., 2010; Wang et al., 2011).

During co-evolution of plant host and the pathogen, plants have developed sophisticated recognition systems, especially the effector-triggered immunity (ETI; Jones and Dangl, 2006). The avirulence (*Avr*) genes from pathogen perceived directly or indirectly by the matching resistance (*R*) genes following the gene-for-gene model, which leads to a rapid and enhanced defense response in the host plant, often including hypersensitive response (HR; Ali and Bakkeren, 2011). *R* gene-mediated recognition of pathogen effectors activates a series of defense signaling cascades. So far, numerous *R* genes have been cloned from many plant species (Liu et al., 2007). The largest class of known R proteins includes a nucleotide-binding site and leucine-rich repeat domains (NBS-LRR proteins; Joshi and Nayak, 2013). A number of *R* genes have been cloned from the model species, *A. thaliana,* and have also been used extensively for answering fundamental questions in molecular plant–microbe interactions, including bacterial, viral, fungal, and oomycete pathogens (Nishimura and Dangl, 2010).

The established interactions between oomycetes and the model plant *A. thaliana* represent an important contribution to the understanding of the oomycete pathogenicity mechanisms (Bozkurt et al., 2012). There have been several examples to use *Arabidopsis* to investigate plant–oomycete pathogen interactions (Koch and Slusarenko, 1990; Holub et al., 1995; Roetschi et al., 2001; Robinson and Cahill, 2003; Daniel and Guest, 2006; Attard et al., 2010; Schlaeppi et al., 2010; Wang et al., 2011, 2013), especially after the whole genome sequence of *Arabidopsis* was announced (*Arabidopsis* Genome Initiative [AGI], 2000). Among the pathosystems established, the best studied model, downy mildew, exhibits extensive variation with *A. thaliana*, which provides a rich resource for identification of at least 27 *RPP* genes (Coates and Beynon, 2010), from which several genes have been cloned using map-based cloning. In contrast, little is known about the *Avr* genes, with only four cloned, including *ATR13* (Allen et al., 2004), *ATR1* (Rehmany et al., 2005), *ATR5* (Bailey et al., 2011), and *ATR39-1* (Goritschnig et al., 2012). In addition, several functional resistance genes from potato and soybean, conferring resistance to *P. infestans* and *P. sojae*, respectively, have also been cloned. Most of the cloned *R* genes against oomycete pathogens belong to the NBS-LRR class of plant resistance genes (Gururani et al., 2012). More identified *R* genes and corresponding *Avr* genes have provided major insights into the mechanism of plant–oomycete interactions (Stassen and Van den Ackerveken, 2011).

However, these studies have been limited to race-specific resistance genes. The race-specific *R* genes are usually difficult to provide long-lived resistance in the field, because the encoded resistance is based on the recognition of corresponding *Avr* genes. For example, all of the 11 *R* genes originated from *Solanum demissum* have lost resistance to *P. infestans* (Song et al., 2003). Broad-spectrum disease resistance, which refers to resistance against different pathogen species or the majority of races of one species, is desirable (Kou and Wang, 2010). However, whether a broad-spectrum resistance gene is durable is still debatable. More than 100 disease-resistance genes have been cloned from different plant species (Liu et al., 2007), and in some cases, several *R* genes confer broad-spectrum resistance. For broad-spectrum disease resistance, the first type is the resistance to two or more different pathogens, and the second one is defined as the resistance to the majority races of the same pathogen. A good example of the first type is the *Arabidopsis R* gene *RPW8,* which confers resistance to two different powdery mildew fungal pathogens, *Erysiphe cruciferarum* UEA1 and *E. cichoracearum* UCSC1 (Xiao et al., 2001). *R* genes of the second type have also been identified (Wang et al., 1996; Büschges et al., 1997). And also, the cloned resistance gene *RB* from *S. bulbocastanum* enables potato highly resistant to all known races of *P. infestans* (Song et al., 2003), a destructive oomycete pathogen. *WRR4* encodes a TIR-NB-LRR (Toll-like/interleukin-1 receptor-nucleotide binding-leucine-rich repeat) protein, confers a dominant, broad-spectrum white rust resistance in *Arabidopsis* accession Columbia to at least four races of *Albugo candida*, and requires expression of the lipase-like defense regulator, *EDS1* (enhanced disease susceptibility 1; Borhan et al., 2008, 2010). However, the mechanisms of broad-spectrum disease resistance are different. For example, the *mlo* resistance is a result of the recessive mutations in the barley *Mlo* locus (Büschges et al., 1997), while in some cases, the durability of *R* genes lie in the fitness cost in pathogen evolution to overcome the resistance (Vera Cruz et al., 2000). *Bs2*-mediated broad-spectrum disease resistance is achieved by recognition of *avrBs2*, an avirulence gene important in the fitness of *Xanthomonas campestris* pv. *vesicatoria* and highly conserved among other *X. campestris* pathovars (Tai et al., 1999). In the case of rice bacterial blight, the disease severity was high in 3 years on the *Xa4* and *Xa10* genotypes, but not on the *Xa7*, as the mutation of *avrXa7* was responsible for both the loss of avirulence function and reduced aggressiveness to rice (Vera Cruz et al., 2000). These results suggested that durability of *R* genes could be predicted according to the fitness or virulence contribution of corresponding avirulence genes.

In agricultural ecosystem, genetic resistance is the most efficient form of protection against pathogens. *R* genes have been successfully used in crop improvement programs in the past and are being continuously exploited. Black shank, caused by *P. parasitica*, which is a destructive disease of tobacco worldwide, damages roots, stems, and leaves at any stages of tobacco growth. There are limited known sources of resistance against the pathogen. For example, in the early stage, the only available source of resistance was derived from Fla 301, which exhibited polygenic resistance. Dominant and monogenic resistance from *Nicotiana plumbaginifolia* Viv (*Php*) and *N. longiflora* Cav (*Phl*) was successfully incorporated into burley and flue-cured tobacco cultivars, respectively (Gutiérrez and Mila, 2007; Antonopoulos et al., 2010). However, cultivars with known resistance resources have failed to provide sufficient control against the pathogen in the field. Thus, there is a need to find new resistance resources. Apart from this, it is highly desirable to understand the plant–pathogen interaction in order to develop novel disease-control strategies and improve disease-control measures.

Little is known about the host specificities in *P. parasitica* although its interaction with tobacco plants follows a gene-for-gene theory (Perrone et al., 2000) and with *A. thaliana* shows natural variation in host specificity (Wang et al., 2011). In this study, we used detached leaf inoculation method to investigate phenotypic and genetic interaction between *A. thaliana* and *P. parasitica*. Based on careful assessment of variation in resistance phenotypes, we describe in this paper details of four interaction phenotypes. The phenotypic variations in *A. thaliana* to *P. parasitica* provide useful resources for better understanding of the interaction between *Phytophthora* and the host plants. We also characterized the resistance of accession Zurich (Zu-1) to *P. parasitica* strain Pp016. Genetic analysis using segregating populations derived from a cross between the resistant accession Zu-1 and the hyper-susceptible accession Landsberg (Ler) show that the resistance is conditioned by a single semi-dominant locus designated *RPPA1^*Zu-1*^* (for Resistance to *P. parasitica 1*). Since *RPPA1^*Zu-1*^* confers resistance to diverse *P. parasitica* strains, its future cloning and analysis will facilitate improved understanding of broad-spectrum disease resistance in plants to oomycete pathogens.

## Materials and Methods

### *Phytophthora parasitica* Culture Conditions and Pathogenicity Assays

The *P. parasitica* strains were routinely cultured on 5% (v/v) cleared carrot juice agar (CA) medium supplemented with 0.002% (w/v) β-sitosterol and 0.01% (w/v) CaCO_3_ in the dark at 23°C. The *P. parasitica* zoospore preparation and pathogenicity assays were as described (Wang et al., 2011). For assessing the phenotype of *Arabidopsis* accessions to *P. parasitica*, at least ten plants were selected (two leaves from each plant) for the pathogenicity assays. The determination of a whole accession as a specific phenotype is based on the infection of leaves of majority plants (more than 70–80%). All experiments were repeated three times.

### *Arabidopsis thaliana* Growing Conditions

The preservation of seeds followed a standard protocol (Martínez-Zapater and Salinas, 1998). For the detached leaf inoculation experiments, the plants were grown in soil at 20–25°C with a photoperiod of 12 h day/night and detached leaves of 28–30 days-old seedlings were used for inoculation. For whole seedling inoculation experiments, the plants were sown on half-strength MS plates and were grown in a chamber at 22°C with a photoperiod of 12 h day/night and 2 weeks-old plants were used for inoculation.

### Microscopic Examination

To visualize the *P. parasitica* infection structures and plant cell death, leaf, and root tissues were harvested at different time points and stained using a modified trypan blue method as described by Wang et al. (2011). The samples were mounted in 50% glycerol and viewed under Olympus BX-51 microscope equipped with differential interference contrast (DIC) optics (Olympus, Japan). For microscopic characterization of infection with *P. parasitica* transformant 1121, which stably expresses green fluorescent protein (GFP), infected tissues were collected and viewed under Olympus BX-51 fluorescent microscope with the GFP filter (BP450–BP480).

### Callose Deposition Staining

To visualize callose deposition, the *A. thaliana* leaf tissues were stained with aniline blue (Bouwmeester et al., 2011). After inoculation, the tissues were cleared overnight in 96% ethanol and stained with 1% (w/v) aniline blue in 150 mM K_2_HPO_4_ (pH 9.5) for 1 h. The samples were mounted on glass microscope slides in 50% glycerol and viewed under Olympus BX-51 fluorescent microscope with the UV filter (BP330–BP385).

### Association Mapping of *RPPA1^Zu-1^* Using SLAF-seq

Two phenotypically contrasting bulks each comprising 30 F_2_ plants, one resistant and the other susceptible to *P. parasitica* were generated. Including the parent pools, we totally sequenced four pools for determination of the regions associated with resistance phenotype. The raw sequencing data have been deposited to the Sequenced Read Archive (Bioproject number: PRJNA282643). The accession numbers for the parent accessions Zu-1 and Ler-1, and the resistant and susceptible bulks are SRR2002811, SRR2002808, SRR2002809, and SRR2002285, respectively. The specific-locus amplified fragment sequencing (SLAF-seq) procedure (Biomarker, Beijing, China) was performed as described (Sun et al., 2013). For genome-wide association analysis, all the markers were parsed as multiple contingency tables and Fisher’s exact tests were used to calculate the associations of the makers with the resistance locus. Multiple tests were controlled by false discovery rate using a Bonferroni procedure as developed in the R program (http://www.r-project.org).

## Results

### Phenotypic Variations in *Arabidopsis thaliana to Phytophthora parasitica* Pp016

In a previous study (Wang et al., 2011), we examined the interaction phenotypes of *A. thaliana* accessions to *P. parasitica* strain Pp016. By using root inoculation method, almost all of the 25 *A. thaliana* accessions were susceptible. However, by infection of detached leaves, some of the 20 briefly examined accessions exhibited varying degrees of resistance. To further investigate the resistance on the leaves of *A. thaliana* response to *P. parasitica*, we examined additional 28 accessions by inoculation of detached leaves with *P. parasitica* Pp016, and examined the extent of disease development macroscopically and microscopically 3 days post inoculation (dpi). The results showed that the 48 (including 20 accessions briefly examined in previous study) accessions (**Table 1**) tested can be categorized into four distinct groups (N, Y, W, H) according to the resistance response, disease severity scored with water-soaked lesion size, development of abundant haustoria, and production of sporangia. The interaction phenotypes were characterized as follows:

**Table 1:** Phenotypic variation of *Arabidopsis thaliana* accessions (48) to *Phytophthora parasitica* strain Pp016.

Phenotypes	Accessions
N	Zu-1^#^
Y	Bla-11, Et-0, Gre-0, Lz-0, Ost-0, Tsu-1 (Di-1, Is-0, Mc-0)^#^
W	Aa-0, Ang-1, Bl-1, Chi-0, Cit-0, Do-0, Fi-0, Ge-0, Gie-0, In-0, Je-0, Mr-0, No-0, Nok-0, Per-2, Rou-0, Rsch-0, Ru-0, Su-0, Tul-0, Uk-1, Zu-0 (Bs-1, Co-1, Col-0, Er-0, Fe-1, Lo-1, Ms-0, Mt-0, Old-1, Pla-2, Sap-0, Sg-1, Sorbo)^#^
H	Ler^#^, Nd-0^#^, Sf-1^#^

*Leaves of 4 weeks-old *A. thaliana* seedlings were assessed for susceptibility by inoculation with *P. parasitica* Pp016 zoospore suspension (1 × 10^5^ zoospores/mL), and the results were scored 3 dpi; ^#^accessions tested in previous study (Wang et al., 2011)*.

**FIGURE 1:**
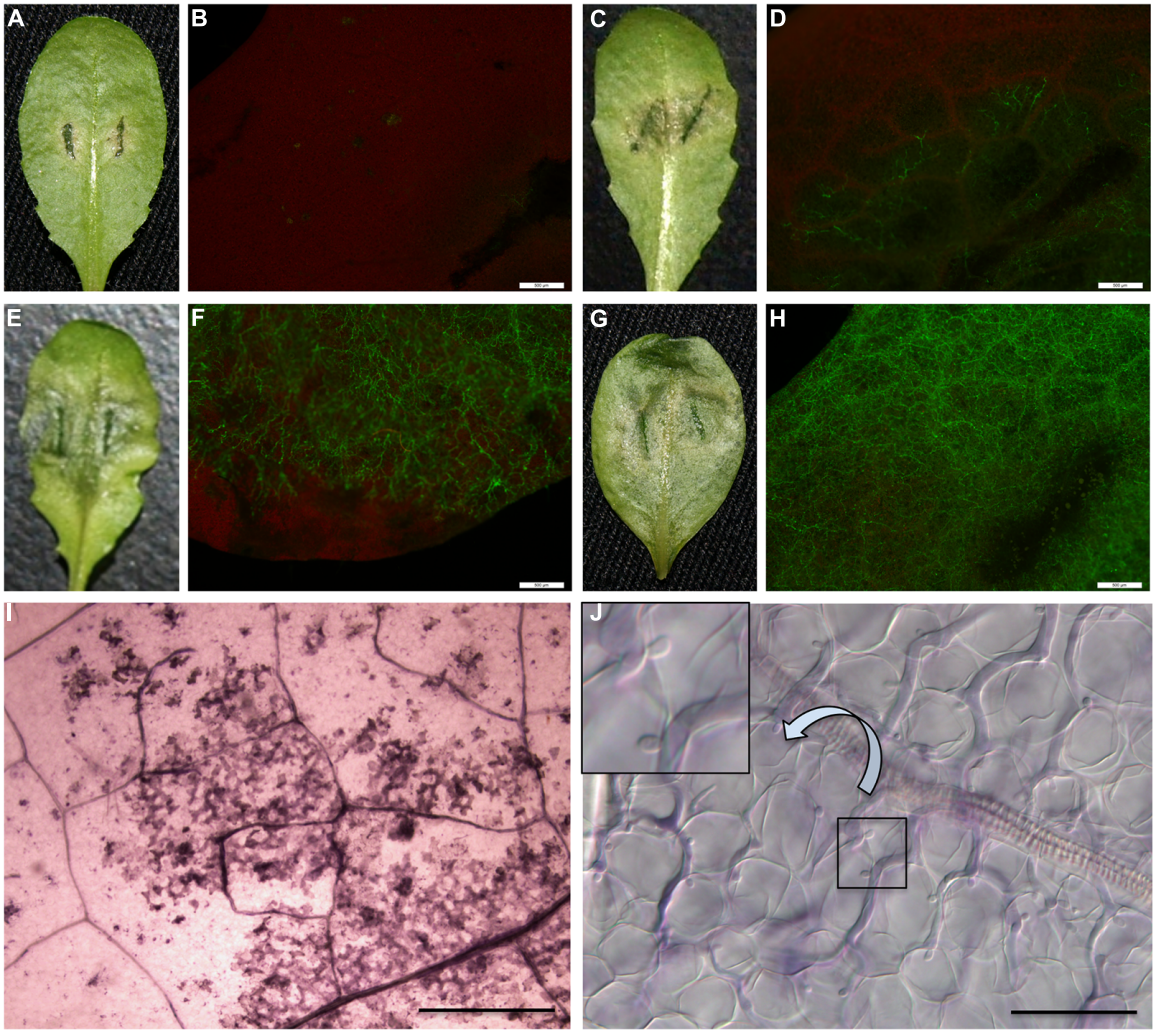
**Variation of interaction phenotypes of *Arabidopsis thaliana* accessions inoculated with *Phytophthora parasitica* strain Pp016**. Leaves of 4 weeks-old *A. thaliana* seedlings were inoculated with zoospores of *P. parasitica* strain Pp016 (1 × 10^5^ zoospores/mL) and the phenotype scored 3 days post inoculation (dpi). To access the extent of pathogen infection, *P. parasitica* transformant 1121 that stably expresses green fluorescent protein (GFP) was used for inoculation. **(A)** Phenotype N, no visible symptom on the leaf surface (Zu-1). **(C)** Phenotype Y, yellowish around the restricted water-soaked lesions that are smaller than one-third leaf sizes (Et-0). **(E)** Phenotype W, water-soaked lesions (Col-0). **(G)** Phenotype H, hyper-susceptible with the whole leaves water-soaked (Ler). **(B,D,F,H)** Cytological characterization of hyphae colonization in **(A,C,E,G)**, respectively, by *P. parasitica* strain 1121 (bar, 500 μm). **(I)** Necrotic flecks were observed under a dissecting microscope at low magnification (4×) (bar, 500 μm). **(J)** Extremely abundant haustoria formed in mesophyll cells in the phenotype H (bar, 50 μm).

Phenotype N (**Figure 1A**): healthy leaves with no visible disease symptoms and nearly no pathogen colonized at the inoculation site (**Figure 1B**), but Necrotic flecks on the leaf surface observed under a dissecting microscope at low magnification (**Figure 1I**). Phenotype Y (**Figure 1C**): Yellowing surrounds inoculation sites within one-third of leaf sizes with spare hyphae (**Figure 1D**) and occasional visible haustoria observed. Phenotype W (**Figure 1E**): Water-soaked lesions on leaves with heavy hyphae colonized (**Figure 1F**) and numerous haustoria formed 3 dpi, and by 4 dpi abundant sporangia formed on the leaf surface as described (Wang et al., 2011). Phenotype H (**Figure 1G**): Hyper-susceptible phenotype exhibited the whole leaves watered with heavier hyphae colonized (**Figure 1H**) and extremely abundant haustoria (**Figure 1J**) formed 3 dpi, and also the sporangia formed earlier than phenotype W on the leaf surface.

Accession Zu-1 exhibited the most resistance to Pp016, and was classified as the phenotype N. The leaves remained green, similar to the control leaves for up to 6 dpi. Nine of 48 accessions were moderately resistant, being classified as the phenotype Y, with restricted water-soaked lesions but smaller than one-third of leaf sizes by 3 dpi. Most *A. thaliana* accessions (35 of 48) were susceptible to Pp016 (scored in this study as the phenotype W), similar to the accession Col-0 as described (Wang et al., 2011). Three accessions, including Ler, were the most susceptible to Pp016 (phenotype H), with whole leaf water-soaked and heavily colonized by 3 dpi.

### Zu-1 is Highly Resistant to a Set of Diverse *P. parasitica* Strains

Of the 48 accessions tested, Zu-1 exhibited the most resistant phenotype to Pp016. To investigate whether the Zu-1 resistance phenotype is broad-spectrum, we performed infection assays with additional 19 *P. parasitica* strains isolated from different host plants (**Table 2**). The results indicated that Zu-1 is highly resistant to all of examined strains. Accession Ler is susceptible to all the strains except Pp008 and Pp009, and hyper-susceptible to a half of strains tested. Accession Col-0 exhibited different phenotypes to the 20 examined *P. parasitica* strains.

**Table 2:** Phenotypic characterization of *A. thaliana* accessions Zu-1, Ler, and Col-0 to 20 *P. parasitica* strains.

*Phytophthora parasitica* strains			*Arabidopsis* accessions		
Strains	Host plant	Mating type	Zu-1	Ler	Col-0
Pp004	Tobacco	A1	N	H	Nd
Pp008	Tobacco	A2	N	N	N
Pp009^#^	Tobacco	A2	N	N	N
Pp010	Pawpaw	A2	N	H	W
Pp012	Pawpaw	A2	N	W	Y
Pp014^#^	Tobacco	A2	N	W	H
Pp016^#^	Tobacco	A1	N	H	W
Pp017	–	A2	N	W	W
Pp018	*Banksia* species	A2	N	H	W
Pp019	–	A2	N	W	Nd
Pp020	*Banksia* species	A2	N	H	Nd
Pp022	*Citrus* species	A1	N	H	Y
Pp023	*Citrus* species	A1	N	W	Y
Pp024	*Citrus* species	A1	N	H	Y
Pp025^#^	*Dendrobium candidum*	A2	N	H	H
Pp026	*D. candidum*	A2	N	H	Nd
Pp028	*D. candidum*	A2	N	W	Nd
Pp029	*D. candidum*	A2	N	W	Nd
Pp030	*D. candidum*	A2	N	W	Nd
Pp031	Tobacco	-	N	H	W

*Leaves of 4 weeks-old *A. thaliana* seedlings were assessed for susceptibility by inoculation with *P. parasitica* zoospore suspension (1 × 10^5^ zoospores/mL), and the results were scored 3 days post inoculation; nd, not determined; ^#^strains used in a previous study (Wang et al., 2011)*.

### Microscopic Characterization of Resistance in Zu-1 to *P. parasitica*

**FIGURE 2:**
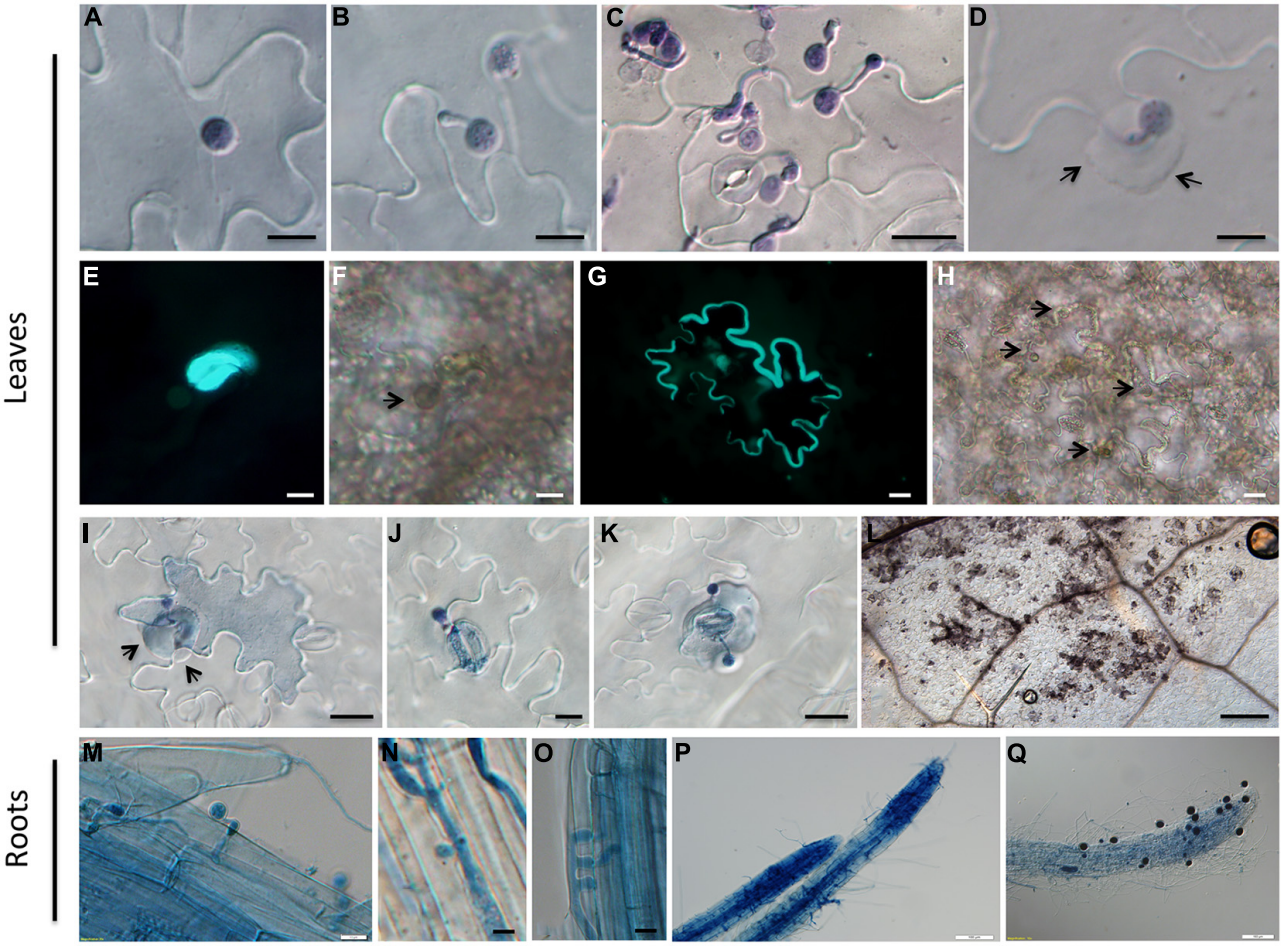
**Cytological characterization of infection of *A. thaliana* accession Zu-1 to *P. parasitica* Pp016. (A)** Zoospores encysted at 30 min (bar, 10 μm). **(B)** Cyst germinated with a germ tube at 1.5 hpi (bar, 10 μm). **(C)** Cyst germinated and appressorium formed at 3 hpi (bar, 20 μm). **(D)** Heavy deposition of materials surrounding the attempted penetration sites (arrows) at the border of two anticlinal walls of epidermal cells (bar, 10 μm). **(E)** Fluorescence of callose at the penetration sites along the junction between epidermal cell walls (bar, 10 μm). **(F)** Bright-field image of **(E)**, showing a germinated cyst at the penetration site (bar, 10 μm). Arrows point to the place where the cyst is visible. **(G)** Fluorescence and thickening of the cell wall showing an infected epidermal cell (bar, 20 μm). **(H)** Bright-field image of **(G)** showing the germinated cysts at the penetration sites (bar, 20 μm). Arrows point to places where the cysts are visible. **(I–K)** Hypersensitive response (HR) of an epidermal cell (bar, 20 μm), stoma guard cells (bar, 10 μm), and an epidermal cell close to the stoma (bar, 20 μm), in response to penetration by Pp016 at 48 hpi. Arrows indicate the heavy deposit of material surrounding the attempted penetration site in epidermal cells. **(L)** Necrosis on the leaf surface 72 hpi and the hypersensitive cells are stained as darker color (bar, 200 μm). **(M)** Zoospores germinated and formed appressoria at 6 hpi (bar, 10 μm). **(N,O)** Haustoria-like structures developed in the cortex at 12 hpi (bar, 10 μm). **(P)** Heavy hyphal colonization in roots at 48 hpi (bar, 100 μm). **(Q)** Sporangia produced on the root surface at 72 hpi (bar, 100 μm).

To further understand the resistance of Zu-1 to *P. parasitica*, we inoculated Zu-1 and the susceptible accession Ler with zoospores of *P. parasitica* strain Pp016 and examined for cellular reactions at different time points microscopically, after the infected tissues were stained with trypan blue. As shown in **Figure 2**, at the initial 3 hours post inoculation (hpi), there were no differences between Zu-1 and Ler in response to *P. parasitica* infection, including the timing of cyst germination, the rates of cyst germination and development of appressoria (**Figures 2A–C**). However, starting from when the penetration pegs emerged beneath the appressoria and the penetration hyphae started to grow between the anticlinal walls of two epidermal cells, differences between Zu-1 and Ler became apparent. In the susceptible Ler plants, the penetration process was similar to Col-0 as described (Wang et al., 2011). However, many more haustoria formed and abundant sporangia produced earlier in Ler than that in Col-0, which is indicative of heavy colonization by strain Pp016. In resistant Zu-1 plants, the earliest microscopically visible response was observed at 6 hpi, when cysts germinated and developed appressoria. At the attempted penetration sites of Zu-1 by Pp016, papilla appeared with heavy deposition of callose materials (**Figures 2D–F**), as revealed by aniline blue staining. Mostly, the wall of some epidermal cells was encased with callose (**Figures 2G,H**). HR is the most important phenotype in the resistant plants against pathogens. Typically, only a few epidermal cells that were penetrated by Pp016 showed a cell death response. This was frequently followed by, at nearly all the infection sites, rapid HRs, which limited in single or a few epidermal cells (**Figure 2I**), or stoma guard cells (**Figure 2J**), or stoma guard cells and an adjacent epidermal cells (**Figure 2K**). This necrotic response typically stops *P. parasitica* infection (**Figure 2L**).

### Zu-1 is Susceptible to Root Infection by *P. parasitica*

Since *P. parasitica* is a typical root pathogen of many plants, we also tested whether Zu-1 is resistant to root infection. Live root tissues were inoculated by dipping into a zoospore suspension. The result showed that Zu-1 is susceptible to *P. parasitica*, with the inoculated seedlings wilted and collapsed 4 dpi. Compared with Ler and Col-0, Zu-1 roots exhibited no clear differences to *P. parasitica* infection. The microscopic characterization showed that the infection process by *P. parasitica* on Zu-1 was similar to that on Ler and Col-0. Zoospores geminated 1.5 hpi and nearly all zoospores developed germ tubes (**Figure 2M**) at 6 hpi. Colonization by invasive hyphae was observed both inside the cells and in the intercellular spaces of the root tissues, and haustoria-like structures (**Figure 2N**) were formed in the cortex at 12 hpi. And in many cases, the haustoria-like structures in roots were as wide as the hyphae they originated from (**Figure 2O**). By 48 hpi, heavy *P. parasitica* hyphae colonization in root tissues was apparent (**Figure 2P**) and, by 72 hpi, numerous sporangia were visible (**Figure 2Q**).

### Genetic Analysis of Resistance in Zu-1 to *P. parasitica*

**FIGURE 3:**
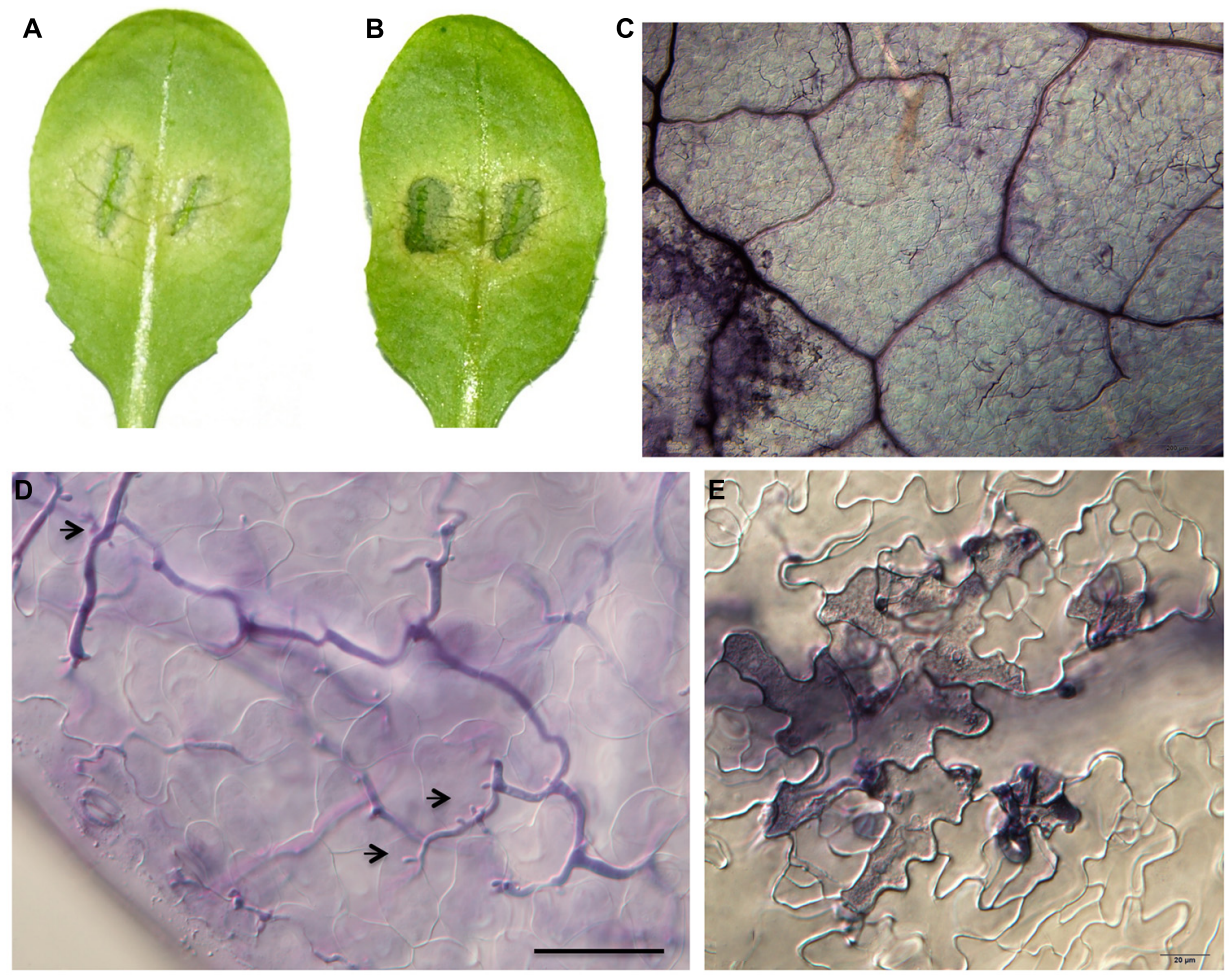
**Phenotypic characterizations of F_1_ progenies of Zu-1 and Ler to infection by *P. parasitica* strain Pp016**. Leaves of 4 weeks-old *A. thaliana* seedlings were inoculated with zoospores of *P. parasitica* strain Pp016 (1 × 10^5^ zoospores/mL) and the phenotype scored 3 dpi. **(A,B)** Leaves of F_1_ progenies are moderately resistant with restricted water-soaked lesions. **(C)** Less colonized hyphae in epidermal cells (bar, 200 μm). **(D)** Less colonized hyphae in mesophyll cells with few haustoria (bar, 50 μm). Arrows indicate haustoria-like structures. **(E)** HR in epidermal cells (bar, 20 μm).

To examine inheritance of resistance in Zu-1, genetic crosses were carried out using Zu-1 and the fully susceptible Ler. The obtained 25 F_1_ plants and the F_2_ populations (**Table 3**) were inoculated with Pp016 and scored for response phenotypes 3 dpi. The results showed that 25 F_1_ progenies exhibited a moderate resistant response (Phenotype Y) with some phenotypic variations to Pp016. Compared with the parents Zu-1 (**Figure 1A**) and Ler (**Figure 1C**), the water-soaked lesions of F_1_ progenies were restricted, generally within (**Figure 3A**) or a little more than one-third of leaf sizes (**Figure 3B**). The surrounding leaf tissues became yellowish 3 dpi, but the colonizing hyphae were restricted within the inoculation sites. The extent of *P. parasitica* colonization in F_1_ progenies with moderately resistant response was also examined microscopically. The invasive hyphae observed were sparse (**Figure 3C**). Compared with abundant haustoria formed in Ler (**Figure 1J**), fewer haustoria were formed in the mesophyll cells from the intercellular penetrating hyphae in F_1_ progenies (**Figure 3D**). Numerous cell death in the epidermal cells also occurred (**Figure 3E**) but not as many as observed for the parent Zu-1, in which nearly all the penetrated epidermal cells responded with cell death.

**Table 3:** Genetic analysis of resistance in *A. thaliana* Zu-1 to *P. parasitica* Pp016.

Plant type	Phenotype	Plants tested	Symptom
Zu-1	N	20	20, N
Ler	H	20	20, H
F_1_	Y (Zu-1 as female)	17	17, Y
	Y (Ler as female)	8	8, Y
F_2_	Segregating	176	42, N
			87, Y
			47, H

Segregation analysis of F_2_ populations showed that one quarter (47/176) were susceptible to Pp016, similar to Ler, while another quarter (42/176) were similar to Zu-1 being resistant to Pp016 (**Table 3**). Nearly half (87/176) of F_2_ populations showed the F_1_ phenotype, being moderately resistant. The segregation (N: Y: S^∗^) ratio was very close to 1: 2: 1 (χ^2^ = 0.307, *P* = 0.8578) in F_2_ populations, consistent with the interpretation that a single semi-dominant locus confers resistance in Zu-1 to *P. parasitica* Pp016. The locus is designated as *RPPA1^*Zu-1*^* (Resistance to *P. parasitica 1*). Also, inoculation of the F_2_ populations with another *P. parasitica* strain Pp025 revealed 100% correlation of phenotypes that was observed with strain Pp016. This indicates that the *RPPA1^*Zu-1*^* locus confers resistance to at least two strains of *P. parasitica*.

### Preliminary Mapping of *RPPA1*^*Zu-1*^ Using SLAF-seq

**FIGURE 4:**
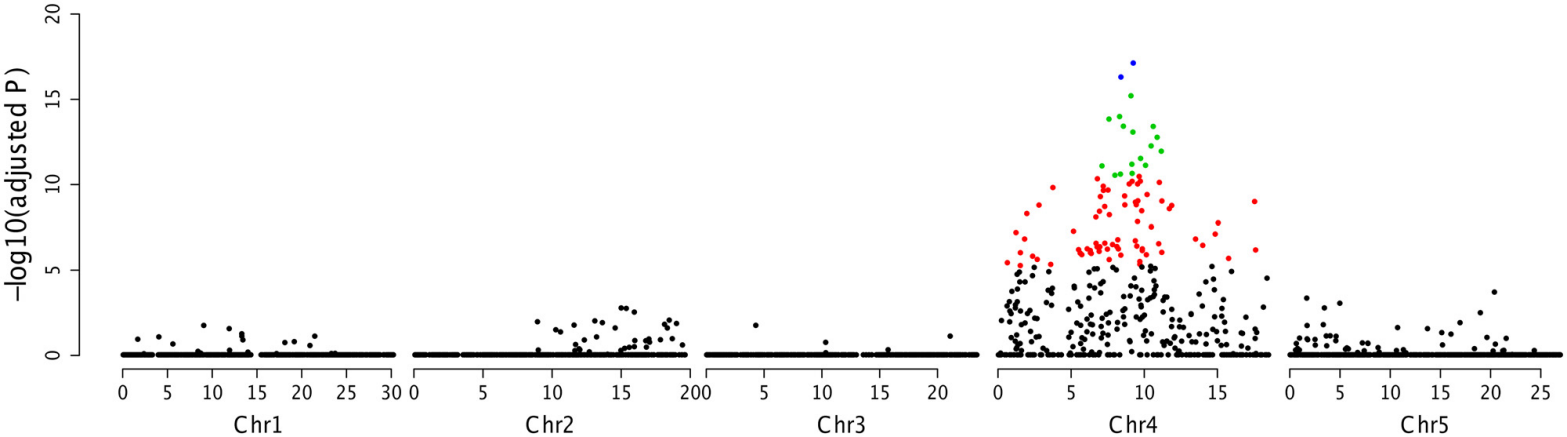
**Association mapping analysis of *RPPA1**^*Zu-1*^*** by SLAF-seq**. The adjusted *P* values for the markers associated with the resistance locus were showed in a logarithmic scale.

By employing SLAF-seq method (Sun et al., 2013), we sequenced four samples, including two parents and two F_2_ bulked populations. As the F_2_ populations were obtained by selfing the F_1_ progeny of a cross between two fully homozygous parents with the genotypes RR or rr, a total of 2742 polymorphic SLAFs were detected and were analyzed for the association with resistance in accession Zu-1 to *P. parasitica* strain Pp016. The results further showed that the strongest associated region was located between 7.1 and 11.2 Mb in the chromosome IV (**Figure 4**).

## Discussion

Elucidation of the mechanism of the interaction between the pathogen and host plants has been the focus for understanding of disease resistance. The objective of this research is to investigate phenotypic and genetic variations in *A. thaliana* to *P. parasitica*. In this paper, we inoculated 28 *A. thaliana* accessions with *P. parasitica* strain Pp016 and variation in resistance phenotypes was evident. Based on careful assessment of variation in resistance phenotypes to *P. parasitica* strain Pp016, the 48 accessions can be divided into four distinct groups. We found that most accessions (38/48) are susceptible to Pp016, including three hyper-susceptible accessions. Nine of 48 accessions are resistant. We also showed that Zu-1 is highly resistant to all 20 strains tested. The genetic analysis of segregating populations derived from cross Zu-1 and Ler showed that the resistance to at least two of *P. parasitica* strains is conditioned by a single semi-dominant locus.

*A. thaliana* accession Zu-1 is resistant to 20 *P. parasitica* strains isolated from different host plants, including tobacco, pawpaw, *Banksia* species, *Citrus* species, and *Dendrobium candidum*. Apart from the origin the *P. parasitica* strains were isolated, the strain selection was also based on their great differences in virulence spectrum on a set of 12 tobacco cultivars (data not shown). For example, strain Pp014 is virulent to all 12 tobacco cultivars, Pp009 is virulent to only three of them, and Pp016 is virulent to eight of them.

Zu-1 was highly resistant to all examined *P. parasitica* strains and microscopically exhibited strong cell death response. Resistance in Zu-1 was manifested as necrosis of plant cells in the epidermal layer (**Figures 2I–K**), which comprised only one or a few epidermal cells, and the pathogen was not observed in the mesophyll cells, which is different from the resistance phenotype of Col-0 in response to infection with *P. parasitica* strain Pp009 (Wang et al., 2011), and other *Phytophthora* pathogens on *A. thaliana* (Roetschi et al., 2001; Wang et al., 2013), in which the mesophyll cells undergoing HR to stop the penetration of progressive hyphae. The diverse range of reaction phenotypes of different plant–pathogen combinations also occurs in *A. thaliana* to the oomycete pathogen *Hyaloperonospora arabidopsidis* (Koch and Slusarenko, 1990; Parker et al., 1993; Reignault et al., 1996). The different responses of known resistance were due to the presence of different resistance loci. The thickening of the cell wall and the formation of callose-containing papillae were evident in Zu-1, which were common characteristics in other plant resistance responses (**Figures 2D–H**). Massive callose depositions were observed at the penetration sites and in the cells undergoing HR (**Figure 2I**). These results suggest that resistance in Zu-1 is triggered at early stage of infection. In addition, of the described *A. thaliana* and *Phytophthora* interactions (Wang et al., 2011, 2013), the failure of the first pathogen penetration often leads to the development of secondary germ tubes and appressoria. Interestingly, we did not observe this phenomenon in Zu-1. Furthermore, at the initial 6 hpi, cyst germination and appressorial development were similar to that on the susceptible Ler.

Zu-1 is susceptible to root infection by *P. parasitica* strain Pp016, which is very different from its highly resistant to leaf infection. Actually, with root inoculation method, most accessions of *A. thaliana* cannot prevent the *P. parasitica* pathogen (Attard et al., 2010; Wang et al., 2011). It is evident that different plant organs may elicit the activation of specific signaling networks. Organ-specificity of defense responses in plant disease has been described (Hermanns et al., 2003; Rookes et al., 2008). A particular pathogen only infects some organs of a genetically susceptible host but not other organs or the entire plant. As described in many plant–pathogen interactions, the plant defense hormones salicylic acid (SA), jasmonic acid (JA), and ethylene (ET) have been shown to play important roles in the resistance reaction. SA-signaling is important for defense against biotrophic pathogens, while JA and/ or ET-signaling is involved in defense against necrotrophic pathogens, although there are exceptions and additional complexities (Glazebrook, 2005). During root infection by *P. parasitica*, SA, JA, and ET signaling pathways cooperate in the defenses (Attard et al., 2010), which is similar with *Arabidopsis* defense response against the root fungal pathogen *Fusarium oxysporum* (Berrocal-Lobo and Molina, 2008), but is different from other *Phytophthora* pathogens on *Arabidopsis* (Roetschi et al., 2001; Rookes et al., 2008; Schlaeppi et al., 2010; Wang et al., 2013). However, most of the results were obtained from the leaf infections. The underlying mechanisms mediating differences of defense pathways in host plants and the organ-specificity are complicated. Zu-1 exhibited obvious differences in resistance to *P. parasitica* and may provide an excellent model for understanding organ-specific disease resistance.

Since semi-dominance is a widespread characteristic of resistance genes and the mechanistic implications have been discussed (Crute and Norwood, 1986). For example, the first resistance gene *RPP5* from *A. thaliana* to *H. arabidopsidis* was identified as a semi-dominant gene, which resulted in resistance in the heterozygote being lower than in the resistant homozygote (Parker et al., 1993, 1997). In our observation, the moderate resistant phenotype of F_1_ plants of Zu-1 and Ler exhibits yellowish characters macroscopically and restricted lesion development. In addition, the F_2_ populations were observed with a ratio very close to 1: 2: 1 (χ^2^ = 0.307, *P* = 0.8578). Based on the phenotype of F_1_ progenies and the F_2_ segregation data, the resistance in Zu-1 to *P. parasitica* Pp016 seemed to be conditioned by a semi-dominant locus, *RPPA1^*Zu-1*^*. However, the heterozygous *RPPA1^*Zu-1*^*in F_2_ plants to *P. parasitica* exhibited intermediate phenotype, making it difficult to achieve fine physical mapping of the *RPPA1^*Zu-1*^* locus. Analysis of selected F_3_ progenies would be necessary to assign accurately a genotype of the plants.

Recent developments in high throughput next-generation sequencing technologies now can provide new strategies for sequence-based genotyping. Several methods have been developed that involve sequencing only a small fraction of the entire genome. Specific-locus amplified fragment sequencing (SLAF-seq) approach is a strategy developed for the *de novo* SNP discovery and genotyping of large populations using an enhanced RRL sequencing method (Sun et al., 2013). And this method has been used for haplotype mapping, genetic mapping, linkage mapping, and polymorphism mapping (Chen et al., 2013; Sun et al., 2013; Zhang et al., 2013; Chen et al., 2014). In this study, we employed the SLAF-seq approach to map the locus *RPPA1^*Zu-1*^*. Association analysis preliminarily determined the *RPPA1^*Zu-1*^* locus in a region between 7.1 and 11.2 Mb in the chromosome IV.

Phenotypic variation was characterized among the interaction with respect to the extent of pathogen colonization and the host response. The timing and degree of pathogen infection and colonization vary among the combinations. HRs range from occasionally in some small regions to almost each infection epidermal cells. The observed phenotypic variations provide a useful resource for investigating molecular process in the interaction between *A. thaliana* and *P. parasitica*. The primary identification and future cloning of the *RPPA1^*Zu-1*^* locus may offer a valuable model for understanding the broad-spectrum resistance in plants against oomycete pathogens.

## Author Contributions

Conceived and designed the experiments: WS; Performed the experiments: YM YH, QZ, and GH; Analyzed the data: WS, YM, QW, and JJ; Contributed reagents/materials/analysis tools: YM, YH, QZ, GH, JQ, and QW; Wrote the paper: YM and WS, with contribution from all authors.

## Conflict of Interest Statement

The authors declare that the research was conducted in the absence of any commercial or financial relationships that could be construed as a potential conflict of interest.
